# Methimazole-Induced Hypothyroidism Increases the Content of Glycogen and Changes the Expression of LDH, GLUT4, and Aromatase in the Pregnant Uterus of Rabbits

**DOI:** 10.3390/metabo15020082

**Published:** 2025-01-30

**Authors:** Marlen Espindola-Lozano, Maribel Méndez-Tepepa, Marlenne Castillo-Romano, Rubicela Rojas-Juárez, Leticia Nicolás-Toledo, Jorge Rodríguez-Antolín, Francisco Castelán, Estela Cuevas-Romero

**Affiliations:** 1 Ph.D. Program in Biological Sciences, Autonomous University of Tlaxcala, 90070 Tlaxcala, Mexico; 20160900@uatx.mx (M.E.-L.); mendezt@uatx.mx (M.M.-T.); 20194308@uatx.mx (R.R.-J.); 2 Master Program in Biological Sciences, Autonomous University of Tlaxcala, 90070 Tlaxcala, Mexico; marnutri01@gmail.com; 3 Center Tlaxcala of Behavior Biology, Autonomous University of Tlaxcala, 90070 Tlaxcala, Mexico; leticia.nicolast@uatx.mx (L.N.-T.); jorge.rodrigueza@uatx.mx (J.R.-A.); fcocastelan@iibiomedicas.unam.mx (F.C.); 4 Department of Cellular and Physiology, Institute of Biomedical Research, Autonomous Nacional University of Mexico, CP 04510 Mexico City, Mexico

**Keywords:** fetus development, estradiol, pregnancy, progesterone, thyroid hormones

## Abstract

**Objective**: To determine the impact of hypothyroidism on uterine glycogen accumulation during pregnancy. **Methods**: Non-pregnant and pregnant (days 5, 10, and 20) rabbits were grouped into control and methimazole (MMI) groups. In rabbits, serum concentrations of thyroxine (T4), triiodothyronine, glucose, insulin, progesterone, and estradiol were quantified. In uterine inter- and implantation sites, the glycogen content and expression of lactate dehydrogenase (LDH), GLUT4, and aromatase were quantified via Western blot. Fetuses’ characteristics at 20 days of pregnancy were analyzed. Two-way ANOVA was used to compare variables between groups. **Results**: Pregnancy reduced T4 concentrations but not T3. In virgin groups, MMI treatment significantly reduced the concentrations of T4 and T3 and increased the expression of GLUT4 and aromatase in the uterus compared to the control group. In pregnant groups, T4, T3, glucose, insulin, progesterone, and estradiol levels were similar between control and MMI-treated rabbits. Compared to controls, MMI treatment in pregnant rabbits (a) reduced GLUT4 expression on inter-implantation sites on day 5; (b) increased glycogen content on implantation sites but reduced GLUT4 expression on inter-and implantation sites on day 10; (c) increased glycogen content and LDH and aromatase expression but reduced GLUT4 on inter-implantation sites; and (d) increased glycogen content and the expression of LDH, GLUT4, and aromatase on day 20 on implantation sites. Moreover, the fetus characteristics were similar between groups. **Conclusions**: MMI-induced hypothyroidism is associated with changes in the uterine content of glycogen and the expression of LDH, GLUT4, and aromatase during pregnancy.

## 1. Introduction

In human and animal models, hypothyroidism is related to infertility, uterine hyperplasia, uterine inflammation, prematurity, spontaneous abortion, preeclampsia, and low birth weight [1,2,3]. In humans, maternal hypothyroidism has been associated with large babies [4] and a high risk of preterm birth and low birth weights [5]. In rats, maternal hypothyroidism induced by thyroidectomy or propylthiouracil reduces the weight of the uterus, placenta, and embryos [6]. Methimazole (MMI) treatment for 50 days in female rabbits also reduces abdominal size in fetuses [3]. Endometrium and fetal tissues contain thyroid hormone transporters, deiodinases, and alpha and beta subtypes of thyroid hormone receptors [7].

Glucose, pyruvate, and lactate are energy sources during implantation, placentation, and embryo development. During pregnancy, glycogen is stored in the uterine glands and used by trophoblast and embryonic tissues, being higher toward implantation and decidualization times [8,9] and toward the end of pregnancy [10]. Hexokinase 1, phosphoglycogen synthase, glycogen synthase, glycogen phosphorylase, glucose-6-phosphate-dehydrogenase, and pyruvate dehydrogenase kinase are also present in the uterus, varying among implantation and inter-implantation sites during the decidualization process [9,11]. The glucose uptake in uterine and placental tissues depends on the different glucose transporters, such as GLUT1, GLUT3, GLUT4, and GLUT8 [12,13]. GLUT1 is increased during placentation and at implantation sites [11]. Lactate regulates immune cell activity in the uterus [14], promotes decidualization, and upregulates GLUT4, GPR81, and VEGF in Ishikawa cells [15], as well as inducing uterine muscle contraction during labor [16]. Lactate and lactate dehydrogenase (LDH) are high in implantation vs. inter-implantation sites during decidualization [11]. Changes in the uterine glycogen content have been related to pre-eclampsia and abortions. Glycogen accumulation in the placenta has been reported in preeclamptic pregnancies [17]. In contrast, placenta, spongiotrophoblast, and glycogen cells are diminished in mice with abortions [18]. In rats, hypothyroidism also reduces the labyrinth zone, spongiotrophoblast, and vascular sinus but increases the area covered by glycogen cells at 18 days of pregnancy [6,19].

The uterine accumulation of glycogen and the expression of GLUT4 are promoted by estradiol but reduced by progesterone [20,21]. Uterine lactate and LDH are also regulated by estradiol and progesterone in mice [11]. In addition, thyroid hormones modulate the synthesis and actions of estrogen in the uterus [22,23] and the expression of their nuclear receptors [1]. The regulation of estradiol and progesterone serum levels by hypothyroidism is controversial and depends on the treatment used to induce this thyroid dysfunction (propylthiouracil or MMI). Thus, both reductions [24] and increases [25] in estrogen levels have been reported, while an increase [25] or a null effect [24] in progesterone levels has been observed.

Hypothyroidism has been related to pregnancy pathologies affecting both dam and progeny health [2,4,5,7]. However, the mechanisms involved in this are unknown. To approach this, we determined the effect of hypothyroidism on serum glucose, insulin, estradiol, and progesterone levels in rabbits. Since hypothyroidism increases the uterine population of glycogen cells during early pregnancy [6,19], we analyzed the amount of glycogen and the expression of LDH, GLUT4, and aromatase (as an indicator of estradiol synthesis) in the uterus of hypothyroid rabbits during implantation (day 5 of pregnancy), placentation (day 10 of pregnancy), and placenta maturation (day 20 of pregnancy) [26,27]. These molecules were quantified and compared in implantation and inter-implantation sites on days 10 and 20 of pregnancy. Although rabbits are polytocous, they have hemochorial-type placentation resembling humans [28].

## 2. Materials and Methods

### 2.1. Animals

**Figure 1:**
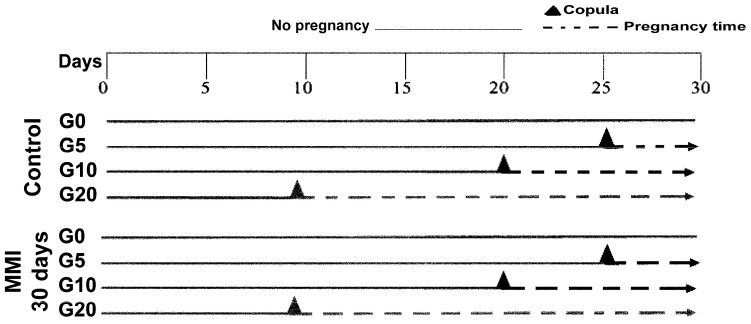
Scheme of experimental design. Control and methimazole (MMI)-treated female rabbits during pregnancy. Eight groups were used: control virgins (C0), control at day 5 of pregnancy (C5), control at day 10 of pregnancy (C10), and control at day 20 of pregnancy (C20), MMI-treated virgins (H0), MMI-treated at day 5 of pregnancy (H5), MMI-treated at day 10 of pregnancy (H10), and MMI-treated at day 20 of pregnancy (H20). All MMI-treated groups underwent 30 days of treatment, with days adjusted to before and after copula for each group. n = 6 per group.

Forty-eight European Chinchilla-breed female rabbits (*Oryctolagus cuniculus* 9–12 months old) were housed in individual steel cages under artificial lighting conditions (16:8 h light–dark and kept at 20 ± 2 °C). Virgin (n = 12) and pregnant rabbits (n = 36) were fed using pellet food chow and had continuous access to drinking water. For each reproductive condition, six rabbits were used as euthyroid, and the other six were hypothyroid, induced through the intake of 10 mg/kg/day of methimazole (MMI) in drinking water for 30 days. This treatment with MMI is effective for inducing hypothyroidism in virgin rabbits [1,29]. Thus, we had eight groups: control virgins (C0; n = 6), control at day 5 of pregnancy (C5; n = 6), control at day 10 of pregnancy (C10; n = 6), and control at day 20 of pregnancy (C20; n = 6), MMI-treated virgins (H0; n = 6), MMI-treated at day 5 of pregnancy (H5; n = 6), MMI-treated at day 10 of pregnancy (H10; n = 6), and MMI-treated at day 20 of pregnancy (H20; n = 6). All MMI-treated rabbits had 30 days of treatment after adjusting days to before and after they were matched (Figure 1). Females copulated four times with expert males to facilitate pregnancy. The day of copulation was considered as day 0 of gestation. Virgin rabbits were fed 120 g of solid food per day. After copula, 300 g of food was provided to pregnant females. Feed was recorded daily, while female body weight was measured weekly. All rabbits used in experimental procedures were euthanized with an overdose of sodium pentobarbital (90 mg/kg; i.p.). The feed was stopped on the day before sacrifice at the end of the experimental period. Blood was obtained from the hearts of rabbits from different groups. The serum was separated and kept at −80 °C until use.

Immediately after death, the right and left uterine horns were excised. For biochemical measures, a portion of the central uterine horns corresponded to the inter-implantation sites (among two implantation sites) and the other corresponded to the implantation sites (including the placenta; only at days 10 and 20 of pregnancy). On day 20 of pregnancy, the number of implantations and reabsorptions in both uterine horns were counted. The fetus’s body weight, the length of the body, and the abdomen diameters were measured in control and MMI-treated females. The protocol was approved by the Ethics Committee of the Universidad Autónoma de Tlaxcala (accepted on 28 August 2015), following the guidelines of the Mexican Law for the Production, Care, and Use of Laboratory Animals.

### 2.2. Hormones, Glucose, and Glycogen Quantification in Dams

In dams from all groups, serum concentrations of total triiodothyronine (T3), thyroxine (T4), insulin, and progesterone were quantified using chemiluminescence by Diagnóstico Molecular y Servicio de Referencia S.A. de C.V. (Diagmo Laboratory, Mexico City, Mexico) [1,29]. The serum concentration of estradiol in dams at 20 days of pregnancy was evaluated using an EIA kit (Cayman Chemical Company, Ann Arbor, MI, USA). Assays were carried out according to the manufacturer’s instructions.

Serum glucose was measured using a kit (ELITech Clinical Systems, ELITechGroup, Paris, France). Portions of left uterine horns (50 mg) from the inter-implantation and implantation sites were disrupted in lysis buffer. Uterine glycogen content was measured with the same kit using a procedure reported elsewhere [1]. Glycogen content in the implantation and inter-implantation sites was calculated as the difference between glycogen chains and free glucose (µmol glucosyl units g^−1^ uterus).

### 2.3. Expression of GLUT4, LDH, and Aromatase

Inter- and implantation sites from the central portion of the left uterine horns (50 mg) were disrupted in lysis buffer, as previously reported [3]. Total protein extracts were obtained via centrifugation at 13,400 rpm for 30 min at 4 °C and quantified afterward. Thus, 30–100 µg of protein was used for the Western blots’ assays depending on the antibody. SDS-PAGE was carried out using 10% acrylamide gels. Later, proteins were transferred to nitrocellulose membranes (BIO-RAD, Hercules, CA, USA, 0.45 µM) and stained with Ponceau’s Red to confirm that protein content was equal in all lines. Subsequently, membranes were blocked with 5–17% non-fat dried milk (depending on the antibody) diluted in 0.02% Tween 20-PBS. Then, they were incubated overnight at 4 °C with antibodies anti-GLUT4 (Santa Cruz Biotechnology, Dallas, TX, USA; sc53566; 1:250), anti-P450 aromatase (Novus Biologicals, Centennial, CO, USA; 1:250; NB100-1596), and anti-LDH (Abcam, Cambridge, UK; 1:200; 98A-1F9BB1. Membranes were incubated for two hours with the secondary antibody 1:250 for GLUT4, 1:1000 for LDH and GLUT4, and 1:20,000 for aromatase. For LDH and aromatase, chemiluminescent signals were detected using a chemiluminescence kit (West Pico Signal, Thermo Fisher Scientific, Waltham, MA, USA) and analyzed with a chemiluminescent signal analyzer (MyECL Imager, Thermo Fisher Scientific). For GLUT4, the signals were captured using photographic plates and Kodak development solutions (developer and mixer) via Kodak GBX Carestream Dental, Rochester, NY, USA. The relative band density for the antigen–antibody complex was calculated using the Image J 14.45S software (National Institute of Health, Bethesda, MD, USA) and normalized to the density of bands covering at least 90% of the length of each Ponceau’s Red-stained Lane [3].

### 2.4. Statistical Analysis

Data are expressed as the mean ± SEM. Two-way ANOVA tests were performed to compare variables between groups considering the time of pregnancy and MMI treatment as factors in the inter-implantation sites. Two-way ANOVA tests were also applied to investigate the impact of hypothyroidism at implantation sites on days 10 and 20 of pregnancy, considering the type of site and MMI treatment as factors. According to normality tests, Student’s *t*- or U-Mann–Whitney tests were carried out to determine significant differences between the fetuses’ characteristics. Values of *p* < 0.05 were considered statistically significant and derived using the GraphPad Prism version 5.01 software.

## 3. Results

**Figure 2:**
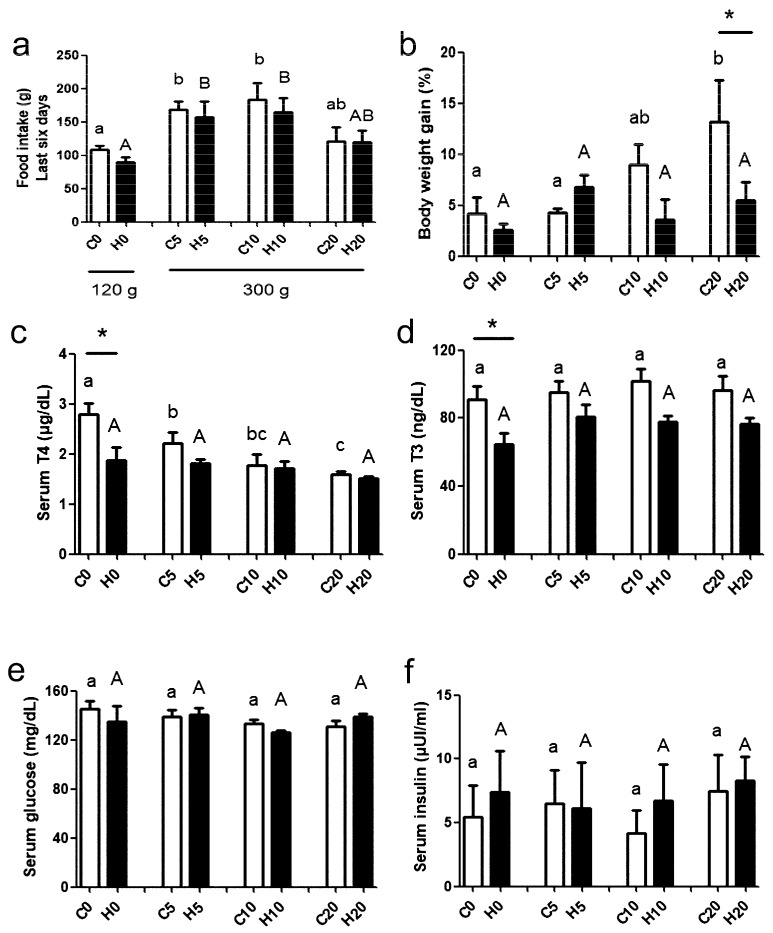
Food intake (**a**), body weight gain (**b**), and serum levels of T4 (**c**), T3 (**d**), glucose (**e**), and insulin (**f**) in virgin (0) and pregnant (5, 10, and 20 days) rabbits grouped into control (C) and MMI-treated (H) groups. Lowercase letters denote control groups and uppercase letters denote MMI-treated groups. Similar letters indicate no statistical significance, and different letters indicate *p* < 0.05. The differences between control and MMI-treated groups are denoted by * *p* < 0.05. Each group had n = 6, except n = 5, which was considered in H5 for insulin and H20 for T4, T3, glucose, and insulin levels. The amount of pellet food disposed of rabbits is shown in (**a**), considering pregnancy conditions.

The food intake of each group during the last six days of treatments was compared. Dams from the two groups ate more food on days 5 and 10 of pregnancy and decreased their food intake at 20 days compared to virgin rabbits (Figure 2a; time of pregnancy, F = 7.5; *p* = 0.0004; MMI treatment, F = 0.9; *p* = 0.33; interaction, F = 0.08; *p* = 0.96). MMI treatment did not modify the significant differences at any time of pregnancy. The body weight gain was significantly higher in control dams on the 20th day of pregnancy compared to the virgin dams and females at day 5 of pregnancy (Figure 2b; time of pregnancy, F = 3.0; *p* = 0.03; MMI treatment, F = 4.7; *p* = 0.03; interaction, F = 2.5; *p* = 0.06). No significant changes were measured in MMI-treated dams at any time of pregnancy. The body weight gain of MMI-treated females was lower than that of the controls when measured on day 20 of pregnancy.

In control dams, serum T4 levels decreased significantly during pregnancy. In MMI-treated dams, the concentration of T4 was similar between H0, H5, H10, and H20. MMI treatment did not lead to significant differences in T4 levels compared to the control, except on day 0 (C0 vs. H0) (Figure 2c; time of pregnancy, F = 7.9; *p* = 0.0003; MMI treatment, F = 8.8; *p* = 0.004; interaction, F = 2.7; *p* = 0.05). The concentration of T3 was stable during pregnancy in both control and MMI-treated dams (Figure 2d; time of pregnancy, F = 1.2; *p* = 0.32; MMI treatment, F = 19.5; *p* < 0.0001; interaction, F = 0.3; *p* = 0.82). However, a significant reduction in the MMI-treated vs. control group was found on day 0. Pregnancy did not change the serum concentration of glucose (Figure 2e; time of pregnancy, F = 1.0; *p* = 0.38; MMI treatment, F = 0.27; *p* = 0.6; interaction, F = 0.4; *p* = 0.72) and insulin (Figure 2f; time of pregnancy, F = 0.2; *p* = 0.84; MMI treatment, F = 0.3; *p* = 0.53; interaction, F = 0.1; *p* = 0.95) in control and MMI-treated rabbits.

**Figure 3:**
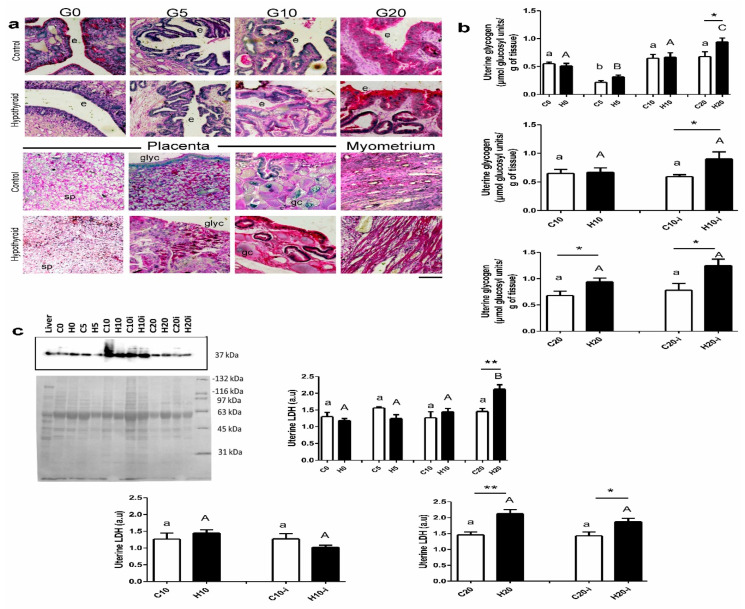
Glycogen content (**a**,**b**) and LDH expression (**c**) in the inter-implantation and implantation (i) sites from the central portion of right uterine horns of the virgin (0) and pregnant (5, 10, and 20 days) rabbits grouped into control (C) and MMI-treated (H) groups. Inter-implantation and implantation sites were considered at days 10 and 20 of pregnancy after placentation occurred. (**a**) Microphotographs of slides stained with periodic acid (PAS) indicating the different uterine tissues in the groups (days 0, 5, 10, and 20) and the placentas on day 20 of pregnancy showing glycogen content. Scale = 100 µm. Abbreviations: epithelium (e), giant cells (gc), spongioblast (sp), and glycogen cells (glyc). The liver was a positive tissue for LDH Western blot (**c**). Two-way ANOVAs were applied for each condition (inter-implantation sites during pregnancy, day 10 of pregnancy in the inter-implantation vs. implantation group, or day 10 of pregnancy in the inter-implantation vs. implantation group). Lowercase letters denote control groups and uppercase letters denote MMI-treated groups. Similar letters indicate no statistical significance, and different letters indicate *p* < 0.05. Differences between control and MMI-treated groups are denoted by * *p* < 0.05 and ** *p* < 0.01. Each group had n = 6, except in C0 for glycogen (n = 5).

Glycogen was identified in diverse cells such as epithelium, stroma, smooth muscle, decidua, giant, and glycogen trophoblast (Figure 3a). In the inter-implantation sites of the control and MMI-treated rabbits, the uterine glycogen content was significantly low on day 5 of pregnancy. The control and MMI-treated groups showed no differences in uterine glycogen content between days 0, 10, and 20 of pregnancy. Compared to control groups, MMI treatment increased the uterine glycogen content on day 20 of pregnancy (Figure 3b; time of pregnancy, F = 33.4; *p* < 0.0001; MMI treatment, F = 4.6; *p* = 0.03; interaction, F = 2.6; *p* = 0.05). On day 10 of pregnancy, the glycogen concentration in the uterus was similar in the inter-implantation and implantation sites in control and MMI-treated dams. In the implantation sites, MMI-treated pregnant rabbits had a higher uterine glycogen content compared to controls (Figure 3b; uterine site, F = 1.0; *p* = 0.30; MMI treatment, F = 4.1; *p* = 0.05; interaction, F = 3.1; *p* = 0.08). On day 20 of pregnancy, the glycogen concentration in the uterus was similar in the inter-implantation and implantation sites in control and MMI-treated dams. In both the inter-implantation and implantation sites, MMI-treated rabbits had a higher uterine glycogen content compared to controls (Figure 3b; uterine site, F = 3.9; *p* = 0.06; MMI treatment, F = 12.5; *p* = 0.002; interaction, F = 1.0; *p* = 0.32).

The LDH expression in the inter-implantation sites did not vary significantly during pregnancy in control rabbits. In MMI-treated groups, a significant increase in the expression of LDH was found on day 20 of pregnancy compared to H0, H5, and H10. On day 20 of pregnancy, the value of LDH in the MMI-treated group was higher than that in the control group (Figure 3c; time of pregnancy, F = 8.9; *p* < 0.0001; MMI treatment, F = 1.5; *p* = 0.21; interaction, F = 7.2; *p* = 0.0005). On day 10 of pregnancy, the glycogen concentration in the uterus was similar in the inter-implantation and implantation sites in control and MMI-treated dams. MMI treatment did not modify the expression of LDH in the inter-implantation and implantation sites (Figure 3c; uterine site, F = 2.6; *p* = 0.11; MMI treatment, F = 0.5; *p* = 0.81; interaction, F = 2.9; *p* = 0.10). On day 20 of pregnancy, the LDH expression in the uterus was similar in the inter-implantation and implantation sites in control and MMI-treated dams. In both the inter-implantation and implantation sites, MMI-treated rabbits showed increased uterine LDH expression compared to controls (Figure 3c; uterine site, F = 1.5; *p* = 0.22; MMI treatment, F = 23.9; *p* < 0.0001; interaction, F = 0.95; *p* = 0.33).

**Figure 4:**
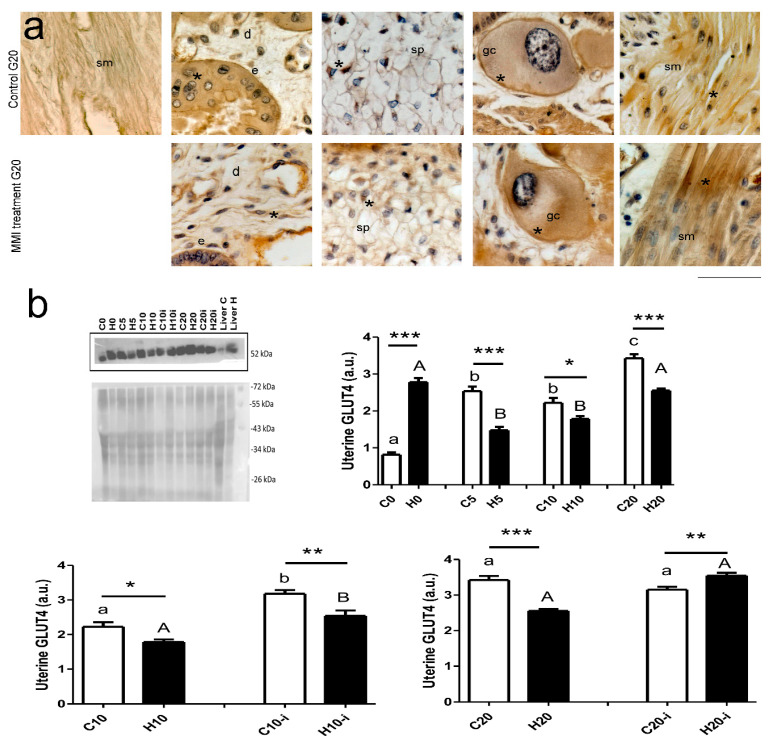
Expression of GLUT4 in inter-implantation and implantation (i) sites from the central portion of right uterine horns of the virgin (0) and pregnant (5, 10, and 20 days) rabbits grouped into control (C) and MMI-treated (H) groups. Inter-implantation and implantation sites were considered at 10 and 20 days of pregnancy. (**a**) Immunohistochemical microphotographs of GLUT4 indicating different tissues in the uterus of dams at 20 days of pregnancy. Positive immunolabeling is indicated by asterisks in pictures. The first picture in the panel indicates the absence of the primary antibody. Scale = 100 µm. Abbreviations: epithelium (e), decidua cells (d), giant cells (gc), spongioblast (sp), and smooth muscle (sm). (**b**) GLUT4 Western blot and Ponceau red-stained membrane in which the liver was a positive tissue (c) Relative quantification of GLUT4 expression. Two-way ANOVA tests were applied for each condition (inter-implantation sites during pregnancy, day 10 of pregnancy in inter-implantation vs. implantation groups, or day 10 of pregnancy in inter-implantation vs. implantation groups). Lowercase letters denote control groups and uppercase letters denote MMI-treated groups. Similar letters indicate no statistical significance, and different letters indicate *p* < 0.05. The differences between control and MMI-treated groups are denoted by * *p* < 0.05, ** *p* < 0.01, and *** *p* < 0.001. Each group had n = 6.

The expression of GLUT4 was identified in different cells, such as epithelium, stroma, smooth muscle, decidua, giant, and glycogen trophoblast (Figure 4a). In inter-implantation sites, the expression of GLUT4 was higher during pregnancy than in virgin females in the control groups. In MMI-treated groups, the expression of GLUT4 was significantly lower at 5 and 10 days of pregnancy compared to virgin dams and those at 10 days of pregnancy. Compared to control groups, the MMI-treated virgin group showed a higher expression of GLUT4, but this was significantly lower at 5, 10, and 20 days (Figure 4b; time of pregnancy, F = 38.0; *p* < 0.0001; MMI treatment, F = 14.3; *p* = 0.0005; interaction, F = 44.8; *p* < 0.0001). On day 10 of pregnancy, the GLUT4 expression in the implantation sites was higher than that in the inter-implantation sites in both control and MMI-treated dams. MMI treatment reduced the expression of GLUT4 in the inter-implantation and implantation sites (Figure 4b; uterine site, F = 53.4; *p* <0.0001; MMI treatment, F = 21.0; *p* = 0.0002; interaction, F = 0.72; *p* = 0.40). On day 20 of pregnancy, the GLUT4 expression in the uterus was similar in the inter-implantation and implantation sites in control and MMI-treated dams. MMI-treated rabbits had a lower uterine GLUT4 expression compared to controls in the inter-implantation site but a higher expression in the implantation site (Figure 4b; uterine site, F = 18.3; *p* = 0.0004; MMI treatment, F = 8.2; *p* = 0.009; interaction, F = 53.9; *p* <0.0001).

**Figure 5:**
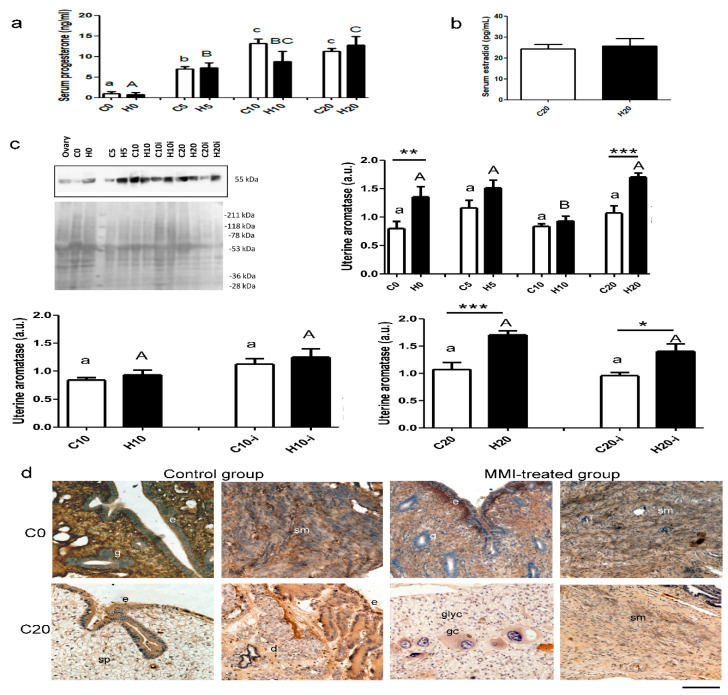
Serum progesterone (**a**), estradiol (**b**) levels, and aromatase expression (**c**) in inter-implantation and implantation (i) sites from the central portion of right uterine horns of virgin (0) and pregnant (5, 10, and 20 days) rabbits grouped into control (C) and MMI-treated (H) groups. Inter-implantation and implantation sites were considered at 10 and 20 days of pregnancy. The ovary was a positive tissue for aromatase Western blot (c). Two-way ANOVAs were applied for each condition (inter-implantation sites during pregnancy, day 10 of pregnancy in inter-implantation vs. implantation sites, or day 10 of pregnancy in inter-implantation vs. implantation sites). Lowercase letters denote control groups and uppercase letters denote MMI-treated groups. Similar letters indicate no statistical significance, and different letters indicate *p* < 0.05. Differences between control and MMI-treated groups are denoted by * *p* < 0.05, ** *p* < 0.01, and *** *p* < 0.001. Each group had n = 6, except in C20 and H20 for estradiol levels and H0 and C10 for aromatase (n = 5). (**d**) Immunohistochemical microphotographs of aromatase indicating positive different tissues in color brown in the uterus of rabbits in each group (days 0, 5, 10, and 20). Scale = 100 µm. Abbreviations: epithelium (e), uterine gland (g), decidua cells (d), giant cells (gc), smooth muscle (sm), spongioblast (sp), and glycogen cells (glyc).

In control groups, the serum concentration of progesterone was significantly higher during pregnancy (days 5, 10, and 20) compared to virgin rabbits. A similar pattern was found in MMI-treated female rabbits on days 5, 10, and 20; these rabbits had higher levels of progesterone than virgin rabbits. No significant differences were observed between control and MMI-treated dams at any time of pregnancy (Figure 5a, pregnancy time, F = 25.3; *p* < 0.0001 MMI treatment, F = 0.66; *p* = 0.42; interaction, F = 1.7; *p* = 0.17). The serum concentration of estradiol on day 20 of pregnancy was unaffected by MMI treatment (U = 12.0; *p* = 1.0; Figure 5b). In inter-implantation sites, the aromatase expression in the uterus was similar between control groups at any time of pregnancy. In contrast, MMI-treated females showed a lower expression of aromatase at day 10 of pregnancy compared to virgin rabbits and those at days 5 and 20 of pregnancy. A significant difference between control and MMI-treated animals was observed at G0 and G20 (Figure 5c; time of pregnancy, F = 7.9; *p* = 0.0003; MMI treatment, F = 23.4; *p* <0.0001; interaction, F = 2.1; *p* = 0.10). On day 10 of pregnancy, the aromatase expression was similar in the inter-implantation and implantation sites in control and MMI-treated dams. MMI treatment did not affect the expression of aromatase in the inter-implantation and implantation sites (Figure 5c; uterine site, F = 8.5; *p* = 0.0008; MMI treatment, F = 1.0; *p* = 0.031; interaction, F = 0.06; *p* = 0.80). On day 20 of pregnancy, the aromatase expression in the uterus was similar in the inter-implantation and implantation sites in control and MMI-treated dams. MMI-treated rabbits had a higher uterine aromatase expression compared to controls in the inter-implantation and implantation sites (Figure 5c; uterine site, F = 4.2; *p* = 0.05; MMI treatment, F = 27.8; *p* < 0.0001; interaction, F = 0.77; *p* = 0.38). Aromatase was localized in the epithelium, stroma cells, smooth muscle cells, decidua cells, spongioblast, giant cells, etc. (Figure 5d).

Control dams on day 20 of pregnancy had 6–10 fetuses, while MMI-treated dams had 9–12 fetuses. The characteristics of fetuses, such as the number of live fetuses, number of resorptions, body weight, length, and abdominal diameter of fetuses from the control and MMI-treated females, were similar (Table 1). We created a distribution histogram of fetuses according to body weight, which led us to consider three groups: slim (<3.4 g), medium (3.4–4.5 g), and heavy (>4.6 g). Therefore, we grouped fetuses according to body weight and compared the media for each group. MMI treatment did not affect the mean values for the percentage of slim, medium, or heavy fetuses compared to the control group (Table 1).

## 4. Discussion

In the present study, the efficacy of MMI treatment for inducing hypothyroidism in virgin rabbits was confirmed by the reduction in T4 and T3 levels compared to the control group. This agrees with previous reports on virgin rabbits [1,29]. During pregnancy, hypothyroidism was also found in MMI-treated groups. However, no significant differences between the pregnant control and MMI-treated groups were observed following pregnancy-induced natural hypothyroidism. This was indicated by the reduction in T4 concentration, as reported in humans [30]. In agreement with these results, rats are found to have a low level of T4 on day 20 of pregnancy [31]. Also, MMI treatment did not modify food intake and body weight gain in female rabbits, suggesting that the proportion of muscle may be replaced by fat mass, as previously reported for hypothyroidism [32]. The serum glucose and insulin concentrations were unaffected by MMI treatment in virgin and pregnant rabbits. Indeed, no modifications in these variables have been previously reported in virgin rabbits [33] or in virgin or pregnant rats [31,34].

In rabbits, GLUT4 expression was detected in the uterine epithelium, stroma, endometrial glands, and myometrium, as in rats [35]. The expression of GLUT4 in the endometrium changes with the menstrual phase, being higher in the follicular phase and lower in the luteal phase [36]. In virgin rabbits, hypothyroidism did not affect the uterine glycogen concentration or the expression of LDH, but it increased the expression of GLUT4 and aromatase. Furthermore, no changes were found in the serum concentrations of progesterone and estradiol due to hypothyroidism. In agreement with this, a null effect of hypothyroidism on these hormones in virgin rabbits was previously reported [29]. Despite this, MMI treatment affects the expression of estrogen and progesterone receptors in the uterus of virgin rabbits [1], suggesting that hypothyroidism affects actions involving uterine estrogen. Hypothyroidism promotes the infiltration of immune cells and hyperplasia in the virgin uterus [1]. In agreement, a high expression of uterine aromatase is related to inflammation, as reported in endometriosis [37]. Thus, the high expression of GLUT4 and aromatase in the uterus of virgin hypothyroid rabbits may be associated with an inflammatory condition.

In pregnant control rabbits, uterine glycogen concentration was low on day 5 of pregnancy (implantation) compared to the rest of the pregnancy. No changes were observed in LDH and aromatase, but a low expression of GLUT4 was observed on days 5 and 10 compared to day 20 of pregnancy. In rabbits, GLUT4 expression was detected in the uterine epithelium, stroma, endometrial glands, myometrium, and decidual zone, as in rats [35]. The aromatase expression was localized in the endometrium and myometrium of rabbits, as in humans [38]. In rodents, glycogen decreases upon implantation and increases during decidualization [9]. Lactate regulates uterine muscle contraction during labor [16]. Uterine glycogen concentration [20] and GLUT4 expression [13] are regulated via estradiol and progesterone. This regulation involves the effects of steroid hormones on insulin actions [21].

During pregnancy, MMI treatment increased the uterine glycogen concentration and the expression of LDH and aromatase but reduced the expression of GLUT4 at the inter- and implantation sites. We also found that serum progesterone levels were increased by pregnancy but unmodified by MMI treatment. On day 20 of pregnancy, the serum estradiol levels were similar between the control and MMI-treated groups. In agreement with our results, an increase in the uterine population of glycogen cells and a high amount of glycogen in the fetal placenta have been reported in thyroidectomized rats [6,34]. The concentration of lactate increases at the end of pregnancy and, according to our results, this may be related to prematurity induced by hypothyroidism [5]. The reduction in GLUT4 in the pregnant uterus of hypothyroid dams could be related to inflammation since a previous report indicated that the pregnant uterus of hypothyroid rabbits has endometrial hyperplasia [3]. In addition, an increase in the expression of inflammation markers like TNFα, IL10, IL6, and HIF1α was reported in the endometrium of pregnant hypothyroid rats [19]. Hypothyroidism also regulates the expression of integrin avß3, leukemia inhibitory factor, and Mucin 1, which are involved in embryo implantation [25] and associated with the expression of VEGF and vascularization of the placenta [6]. In agreement, a reduction in the endometrial expression of GLUT4 has also been reported in obese patients with polycystic ovary syndrome (PCOS), associated with high levels of TNF-α [39].

The possible inflammation induced by hypothyroidism in the uterus of virgin and pregnant rabbits, which modifies the concentration of glycogen and the expression of LDH, GLUT4, and aromatase, could have consequences for the progeny. In the present study (until day 20 of pregnancy), no differences were found in the number of implantations, reabsorptions, or fetal morphometry. We previously reported that prolonged treatment with MMI (50 days) reduces the size of fetuses [3]. As day 20 of pregnancy, for rabbits, is the end of the second trimester of pregnancy, we do not know whether hypothyroidism could affect embryo development. This is a limitation of this study. Thus, it is necessary to analyze the body weight at birth. In this regard, the consequences of changes in the glycogen reserve and estradiol could be larger babies [4], a high risk of preterm birth, including low birth weight [5], or even alterations in organogenesis and neural development [40].

Another limitation of the present study is that we did not measure the concentration of estradiol and progesterone directly in the uterine tissues. Also, we do not know the sex of each analyzed fetus. Female and male fetuses could have different morphometric characteristics. In contrast, a strength of this investigation is that it shows that hypothyroidism can modify the metabolic and hormonal environment in both implantation and inter-implantation sites in animal models in the first and second trimesters of pregnancy. This study could provide a basis for future studies researching metabolic changes in implantation sites that could affect implantation, placentation, and embryo development.

In conclusion, our results suggest that hypothyroidism may affect glucose uptake in the pregnant uterus, which is associated with the local synthesis of estradiol, as can be inferred by the differences in the expression of aromatase depending on the stage of pregnancy.

## Figures and Tables

**Table 1:** Characteristics of fetuses from control and MMI-treated dams after 20 days of pregnancy.

	Control n = 6	MMI-Treated n = 6	Statistic
Both uterine horns			
Range of implants (min–max)	6–10	9–12	
Number of fetuses per dam	8.2 ± 0.7	9.8 ± 0.5	*t* = 1.78; *p* = 0.09
Number of fetal resorptions per dam	0.3 ± 0.3	0.2 ± 0.2	U = 17.5; *p* = 1.0
Body weight of fetuses (g)	4.0 ± 0.3	4.4 ± 0.2	*t* = 1.02; *p* = 0.33
Abdominal diameter of fetuses (mm)	13.7 ± 0.5	14.1 ± 0.1	*t* = 0.76; *p* = 0.46
Body length of fetuses (mm)	39.2 ± 1.9	42.0 ± 0.8	*t* = 1.31; *p* = 0.21
	48 fetuses	59 fetuses	
% of slim fetuses (<3.4 g)	10.7 ± 7.5	5.0 ± 3.6	U = 17.0; *p* = 0.92
% of medium fetuses (3.4–4.5 g)	62.8 ± 14.9	57.9 ± 11.6	*t* = 0.25; *p* = 0.80
% of heavy fetuses (>4.6 g)	26.3 ± 16.7	36.9 ± 13.8	U = 14.0; *p* = 0.56

## Data Availability

The data presented in this study are available on request from the corresponding author.

## References

[1] Rodríguez-Castelán J., Del Moral-Morales A., Piña-Medina A.G., Zepeda-Pérez D., Castillo-Romano M., Méndez-Tepepa M., Espindola-Lozano M., Camacho-Arroyo I., Cuevas-Romero E. (2019). Hypothyroidism Induces Uterine Hyperplasia and Inflammation Related to Sex Hormone Receptors Expression in Virgin Rabbits. Life Sci..

[2] Brown E.D.L., Obeng-Gyasi B., Hall J.E., Shekhar S. (2023). The Thyroid Hormone Axis and Female Reproduction. Int. J. Mol. Sci..

[3] Rodríguez-Castelán J., Zepeda-Pérez D., Méndez-Tepepa M., Castillo-Romano M., Espíndola-Lozano M., Anaya-Hernández A., Berbel P., Cuevas-Romero E. (2018). Hypothyroidism Alters the Uterine Lipid Levels in Pregnant Rabbits and Affects the Fetal Size. Endocr. Metab. Immune Disord. Drug Targets.

[4] Andersen S.L., Olsen J., Wu C.S., Laurberg P. (2013). Low Birth Weight in Children Born to Mothers with Hyperthyroidism and High Birth Weight in Hypothyroidism, Whereas Preterm Birth Is Common in Both Conditions: A Danish National Hospital Register Study. Eur. Thyroid J..

[5] Pande A., Anjankar A. (2023). A Narrative Review on the Effect of Maternal Hypothyroidism on Fetal Development. Cureus.

[6] Serakides R., Silva J.F., Vidigal P.N., Galvo D.D., Boeloni J.N., Nunes P.P., Ocarino N.M., Nascimento E.F. (2012). Fetal Growth Restriction in Hypothyroidism Is Associated with Changes in Proliferative Activity, Apoptosis and Vascularisation of the Placenta. Reprod. Fertil. Dev..

[7] Colicchia M., Campagnolo L., Baldini E., Ulisse S., Valensise H., Moretti C. (2014). Molecular Basis of Thyrotropin and Thyroid Hormone Action during Implantation and Early Development. Hum. Reprod. Update.

[8] Brison D.R., Leese H.J. (1991). Energy Metabolism in Late Preimplantation Rat Embryos. J. Reprod. Fertil..

[9] Chen Z., Sandoval K., Dean M. (2022). Endometrial Glycogen Metabolism during Early Pregnancy in Mice. Mol. Reprod. Dev..

[10] Vasilenko P., Adams W.C., Frieden E.H. (1981). Uterine Size and Glycogen Content in Cycling and Pregnant Rats: Influence of Relaxin. Biol. Reprod..

[11] Zuo R.J., Gu X.W., Qi Q.R., Wang T.S., Zhao X.Y., Liu J.L., Yang Z.M. (2015). Warburg-like Glycolysis and Lactate Shuttle in Mouse Decidua during Early Pregnancy. J. Biol. Chem..

[12] Vrhovac Madunić I., Karin-Kujundžić V., Madunić J., Šola I.M., Šerman L. (2021). Endometrial Glucose Transporters in Health and Disease. Front. Cell Dev. Biol..

[13] Long Y., Wang Y.C., Yuan D.Z., Dai X.H., Liao L.C., Zhang X.Q., Zhang L.X., Ma Y.D., Lei Y., Cui Z.H. (2021). GLUT4 in Mouse Endometrial Epithelium: Roles in Embryonic Development and Implantation. Front. Physiol..

[14] Gardner D.K. (2015). Lactate Production by the Mammalian Blastocyst: Manipulating the Microenvironment for Uterine Implantation and Invasion?. Bioessays.

[15] Gurner K.H., Evans J., Hutchison J.C., Harvey A.J., Gardner D.K. (2022). A Microenvironment of High Lactate and Low PH Created by the Blastocyst Promotes Endometrial Receptivity and Implantation. Reprod. Biomed. Online.

[16] Madaan A., Nadeau-Vallée M., Rivera J.C., Obari D., Hou X., Sierra E.M., Girard S., Olson D.M., Chemtob S. (2017). Lactate Produced during Labor Modulates Uterine Inflammation via GPR81 (HCA1). Am. J. Obstet. Gynecol..

[17] Burton G.J., Scioscia M., Rademacher T.W. (2011). Endometrial Secretions: Creating a Stimulatory Microenvironment within the Human Early Placenta and Implications for the Aetiopathogenesis of Preeclampsia. J. Reprod. Immunol..

[18] Hosseini M.S., Ali-Hassanzadeh M., Nadimi E., Karbalay-Doust S., Noorafshan A., Gharesi-Fard B. (2020). Stereological Study of the Placental Structure in Abortion-Prone Mice Model (CBA/J×DBA/2J). Ann. Anat..

[19] Santos B.R., dos Anjos Cordeiro J.M., Santos L.C., Barbosa E.M., Mendonça L.D., Santos E.O., de Macedo I.O., de Lavor M.S.L., Szawka R.E., Serakides R. (2022). Kisspeptin Treatment Improves Fetal-Placental Development and Blocks Placental Oxidative Damage Caused by Maternal Hypothyroidism in an Experimental Rat Model. Front. Endocrinol..

[20] Bowman K., Rose J. (2017). Estradiol Stimulates Glycogen Synthesis Whereas Progesterone Promotes Glycogen Catabolism in the Uterus of the American Mink (Neovison Vison). Anim. Sci. J..

[21] Hodonu A., Escobar M., Beach L., Hunt J., Rose J. (2019). Glycogen Metabolism in Mink Uterine Epithelial Cells and Its Regulation by Estradiol, Progesterone and Insulin. Theriogenology.

[22] Steinsapir J., Rojas A.M., Mena M., Tchernitchin A.N. (1982). Effects of Thyroid Hormone on some Uterine Responses to Estrogen. Endocrinology.

[23] Gardners R.M., Kirkland J.L., Ireland J.S., Stancel G.M. (1978). Regulation of the Uterine Response to Estrogen by Thyroid Hormone. Endocrinology.

[24] Kowalczyk-Zieba I., Staszkiewicz-Chodor J., Boruszewska D., Lukaszuk K., Jaworska J., Woclawek-Potocka I. (2021). Hypothyroidism Affects Uterine Function via the Modulation of Prostaglandin Signaling. Animals.

[25] Erbaş E., Gedikli S. (2022). Investigation of the Endometrial Receptivity Status in Experimental Hypothyroid-Induced Female Rats. Iran. J. Basic Med. Sci..

[26] Fischer B., Chavatte-Palmer P., Viebahn C., Santos A.N., Duranthon V. (2012). Rabbit as a Reproductive Model for Human Health. Reproduction.

[27] Krusche C.A., Vloet T.D., Herrler A., Black S., Beier H.M. (2002). Functional and Structural Regression of the Rabbit Corpus Luteum Is Associated with Altered Luteal Immune Cell Phenotypes and Cytokine Expression Patterns. Histochem. Cell Biol..

[28] Chavatte-Palmer P., Tarrade A., Rousseau-Ralliard D. (2016). Diet before and during Pregnancy and Offspring Health: The Importance of Animal Models and What Can Be Learned from Them. Int. J. Environ. Res. Public Health.

[29] Anaya-Hernández A., Rodríguez-Castelán J., Nicolás L., Martínez-Gómez M., Jiménez-Estrada I., Castelán F., Cuevas E. (2015). Hypothyroidism Affects Differentially the Cell Size of Epithelial Cells among Oviductal Regions of Rabbits. Reprod. Domest. Anim..

[30] Yap Y.W., Onyekwelu E., Alam U. (2023). Thyroid Disease in Pregnancy. Clin. Med..

[31] Kent N.L., Atluri S.C., Cuffe J.S.M. (2022). Maternal Hypothyroidism in Rats Reduces Placental Lactogen, Lowers Insulin Levels, and Causes Glucose Intolerance. Endocrinology.

[32] Tsou M.T. (2021). Subclinical Hypothyroidism Represents Visceral Adipose Indices, Especially in Women With Cardiovascular Risk. J. Endocr. Soc..

[33] Rodríguez-Castelán J., Nicolás L., Morimoto S., Cuevas E. (2015). The Langerhans Islet Cells of Female Rabbits Are Differentially Affected by Hypothyroidism Depending on the Islet Size. Endocrine.

[34] Pickard M.R., Leonard A.J., Ogilvie L.M., Edwards P.R., Evans I.M., Sinha A.K., Ekins R.P. (2003). Maternal Hypothyroidism in the Rat Influences Placental and Liver Glycogen Stores: Fetal Growth Retardation near Term Is Unrelated to Maternal and Placental Glucose Metabolic Compromise. J. Endocrinol..

[35] Korgun E.T., Demir R., Hammer A., Dohr G., Desoye G., Skofitsch G., Hahn T. (2001). Glucose Transporter Expression in Rat Embryo and Uterus during Decidualization, Implantation, and Early Postimplantation. Biol. Reprod..

[36] Cui P., Li X., Wang X., Feng Y., Lin J.F., Billig H., Shao R. (2015). Lack of Cyclical Fluctuations of Endometrial GLUT4 Expression in Women with Polycystic Ovary Syndrome: Evidence for Direct Regulation of GLUT4 by Steroid Hormones. BBA Clin..

[37] Bukulmez O., Hardy D.B., Carr B.R., Word R.A., Mendelson C.R. (2008). Inflammatory Status Influences Aromatase and Steroid Receptor Expression in Endometriosis. Endocrinology.

[38] Knapp P., Chabowski A., Błachnio-Zabielska A., Walentowicz-Sadłecka M., Grabiec M., Knapp P.A. (2013). Expression of Estrogen Receptors (α, β), Cyclooxygenase-2 and Aromatase in Normal Endometrium and Endometrioid Cancer of Uterus. Adv. Med. Sci..

[39] Ha L.X., Wu Y.Y., Yin T., Yuan Y.Y., Du Y.D. (2021). Effect of TNF-Alpha on Endometrial Glucose Transporter-4 Expression in Patients with Polycystic Ovary Syndrome through Nuclear Factor-Kappa B Signaling Pathway Activation. J. Physiol. Pharmacol..

[40] Cuevas E., Ausó E., Telefont M., Morreale De Escobar G., Sotelo C., Berbel P. (2005). Transient Maternal Hypothyroxinemia at Onset of Corticogenesis Alters Tangential Migration of Medial Ganglionic Eminence-Derived Neurons. Eur. J. Neurosci..

